# The bone conduction implant BONEBRIDGE increases quality of life and social life satisfaction

**DOI:** 10.1007/s00405-022-07384-w

**Published:** 2022-05-06

**Authors:** C. Irmer, S. Volkenstein, S. Dazert, A. Neumann

**Affiliations:** 1 Department of Otorhinolaryngology, Head and Neck Surgery, St. Elisabeth-Hospital, Ruhr-University Bochum, Bleichstr. 15, 44787 Bochum, Germany; 2 Department of Otorhinolaryngology, Head and Neck Surgery, Rheinlandklinikum Neuss GmbH, Neuss, Germany

**Keywords:** Bonebridge, Bone conduction implant, Hearing rehabilitation, Quality of life

## Abstract

**Purpose:**

Transcutaneous active bone conduction hearing aids represent an alternative approach to middle ear surgery and conventional hearing aids for patients with conductive or mixed hearing loss. The aim of this study was to determine quality of life, subjective hearing experience and patients’ satisfaction after implantation of a bone conduction hearing aid.

**Methods:**

This monocentric and retrospective study included twelve adult patients who received a bone conduction hearing aid (Bonebridge, MedEL) consisting of an extracorporeal audio processor and a bone conduction implant (BCI) between 2013 and 2017. On average 40 months after implantation, the patients were asked to answer three questionnaires regarding quality of life (AqoL-8D), self-reported auditory disability (SSQ-12-B) and user’s satisfaction (APSQ) after implantation of the Bonebridge (BB). A descriptive statistical analysis of the questionnaires followed.

**Results:**

12 patients aged 26–85 years (sex: m = 7, w = 5) were recruited. The quality of life of all patients after implantation of the BB (AqoL 8D) averaged an overall utility score of 0.76 (SD ± 0.17). The mean for ‘speech hearing’ in the SSQ-12-B was + 2.43 (SD ± 2.03), + 1.94 (SD ± 1.48) for ‘spatial hearing’ and + 2.28 (SD ± 2.32) for ‘qualities of hearing’. 11 out of 12 patients reported an improvement in their overall hearing. The APSQ score for the subsection ‘wearing comfort’ was 3.50 (SD ± 0.87), ‘social life’ attained a mean of 4.17 (SD ± 1.06). The ‘device inconveniences’ reached 4.02 (SD ± 0.71) and ‘usability’ of the device was measured at 4.23 (SD ± 1.06). The average wearing time of the audio processor in the cohort was 11 h per day, with 8 of 12 patients reporting the maximum length of 12 h per day.

**Conclusion:**

BB implantation results in a gain in the perceived quality of life (AqoL 8D). The SSQ-12-B shows an improvement in subjective hearing. According to the APSQ, it can be assumed that the BB audio processor, although in an extracorporeal position, is rated as a useful instrument with positive impact on social life. The majority stated that they had subjectively benefited from BB implantation and that there were no significant physical or sensory limitations after implantation.

## Introduction

Hypoacusis or hearing loss is defined as functional deficit of auditory capacity in comparison to a healthy person. It often leads to deterioration in quality of life [1] characterized by social isolation, loneliness as well as stigmatization and is associated with cognitive decline [2–4]. Worsening dementia and depression have been reported as a consequence of hearing loss [5]. According to the WHO, 5% (approx. 430 million) of the world population suffered from hearing loss in April 2021. A further increase to over 700 million affected people in 2050 is predicted [2]. There are indications for hearing rehabilitation with an implantable hearing device in patients who cannot be fitted with a conventional hearing aid due to either medical limitations or unsatisfactory audiological results and in patients with contraindications or unpromising outcome of reconstructive middle ear surgery. Furthermore, there should be an expected long-term improvement in hearing by an implantable hearing system [6, 7].

The success of these devices depends on the audiological hearing improvement on the one hand [8–10] as well as on the patient's compliance on the other hand [9, 10]. Compliance can be influenced, among other things, by the patient’s willingness to take certain measures [11]. The willingness to wear a hearing aid in general thus determines the therapy adherence and directly affects the therapy success [9, 10]. Therefore, the aim of device development should be to maximize both the perceived quality of life and the patient’s satisfaction with the hearing aid.

The partially implantable active bone conduction hearing device Bonebridge (BB) by MedEL (Innsbruck, Austria) consisting of an extracorporeal audio processor and the internal bone conduction implant (BCI) is indicated for patients with conductive hearing loss, mixed hearing loss and single sided deafness (SSD). The evaluation of quality of life and patient’s satisfaction as well as the self-reported auditory disability after implantation was aimed to be determined in this study.

## Material and methods

### Study design

This monocentric, retrospective, observational study was approved by the local ethics committee (Ärztekammer Nordrhein; No. 2017044). The user experience with the BB was reviewed by using three questionnaires.

24 Patients who received the BB between January 2013 and December 2017 were assessed for eligibility. Inclusion criteria for this study was a minimum of three months experience with the BB after activation. Two patients were excluded due to chronic diseases (trisomy 21 and CHARGE syndrome) which would have significantly affected the quality-of-life outcome. Another 2 patients were excluded due to a language barrier. 20 patients were eligible, whereas 8 patients did not consent, and 12 patients finally participated in this study.

On average, the patients were interviewed 40 months (min 8 months; max 68 months) after surgery. The quality of treatment was intended to be surveyed with an emphasis on the BB user’s subjective experience. Therefore, three different questionnaires were used to measure the patient related outcome (PRO) [12].

Patients were asked to answer the Assessment of quality of life (AQoL-8D) questionnaire, the Speech, spatial and qualities of hearing questionnaire (SSQ-12-B) related to self-reported auditory disability and the audio processor satisfaction questionnaire (APSQ) for user satisfaction with the audio processor. No time limit was imposed on the patients to answer the questionnaires. Table 1 shows the patient characteristics (gender, age, implant side), the summarized medical history, the time span between implantation and answering the questionnaires and the bone conduction thresholds of the implanted and contralateral ear. The general quality of life after implantation of the BB was surveyed using 35 questions from the AQoL-8D-questionnaire (Version 12, 2017) [13]. Patients were asked to compare the present situation with the BB to the situation before this hearing rehabilitation. The AQoL consists of health-related multi-attribute utility quality of life instruments represented in 8 variable response dimensions. All dimensions may be scored separately or summarized in two superdimensions (superdimension ‘mental’ utility score calculated from the dimensions ‘mental health’, ‘happiness’, ‘self worth’, ‘coping’ and ‘relationships’ and superdimension ‘physical’ utility score calculated from the dimensions ‘independent living’, ‘senses’ and ‘pain’). The dimension ‘senses’ is composed of the items ‘vision’, ‘hearing’ and ‘communication’. The overall utility score of the AQoL-8D-questionnaire is composed of the superdimension scores [14].

**Table 1:** Patient characteristics

ID	Age (years)	Sex (m/f)	Implanted side	Time from Implantation to questionnaire (months)	Medical history	Bone conduction threshold mean (decibel)	
						Right ear	Left ear
1	45	m	Left	8	History of tympanoplasty and cholesteatoma, conductive hl	15	20
2	81	m	Right	66	History of tympanoplasty and radical cavity, mixed hl	30	25
3	56	m	Left	18	History of tympanoplasty, recurrent auricular perichondritis, mixed hl	25	25
4	27	m	Left	64	History of tympanoplasty and cholesteatoma, mixed hl	30	35
5	26	f	Right	11	Malformation (atresia of external auditory canal), conductive hl	15	5
6	39	m	bilateral	46	Bilateral history of tympanoplasty and cholesteatoma, conductive hl	15	10
7	55	m	Right	68	History of tympanoplasty, cholesteatoma and radical cavity, mixed hl	30	30
8	80	f	Right	44	History of otosclerosis, stapes surgery years ago, mixed hl	35	15
9	83	f	Left	39	History of otosclerosis, stapes surgery years ago- vertigo after surgery, mixed hl	30	35
10	60	f	Right	33	History of tympanoplasty, recurrent external otitis, conductive hl	10	10
11	85	f	Left	54	History of tympanoplasty, mixed hl	25	35
12	47	m	Right	35	Mixed hl (functional deafness), cros with conventional hearing aids not tolerated	65	5

HL—hearing loss, m—male, f—female, CROS—contralateral routing of signals

The comparative self-reported auditory disability before and after BB was evaluated using the SSQ-12-B questionnaire (12 questions) by Noble et al. (Short version B, year 2013) [15]. It includes hearing speech in a variety of contexts (subsection ‘speech hearing’), spatial hearing (subsection ‘spatial hearing’), listening to speech streams with background noise, the ease of listening and the naturalness, clarity and the ability to differ sounds, speakers, musical pieces and instruments (subsection ‘qualities of hearing’) [15, 16].

The APSQ by Billinger-Finke et al. (2020) [17] was developed to assess user satisfaction with an audio processor, which is primarily perceived by patients as the actual hearing aid. A total score and subscale scores for ‘wearing comfort’, ‘social life’, ‘usability’ and ‘device inconveniences’ can be calculated by 21 questions represented in a Likert scale. In addition, the test includes a survey of the wearing time per day and night in hours [17].

### The active bone conduction hearing implant

The BB by MedEl (Innsbruck, Austria) is a partially implantable active bone conduction implant system consisting of an extracorporeal audio processor and a BCI. The audio processor Amadé or Samba of MedEl (Innsbruck, Austria) are fixed over the implant on intact skin by a holding magnet. The BCI by itself contains a receiver coil, a demodulator, and a transducer (Boneconduction-Floating Mass Transducer; BC-FMT). The BC-FMT can be surgically positioned either in the mastoid bone or retrosigmoidally, depending on the specific morphology of the patient‘s skull. For this purpose, the patients received a high-resolution temporal bone computertomography preoperatively to plan the procedure.

### Surgery

Surgery was performed by the senior author in all cases under general anesthesia in a supine position. The mastoid and temporal bone were exposed via a retroauricular approach. The implant bed was milled out to accommodate the BC-FMT. Finally, the implant was inserted and fixed with cortical screws, followed by wound closure in layers. After completion of the primary postoperative healing process, the initial fitting of the audio processor took place 6 weeks later as an outpatient procedure.

### Statistics

Baseline data collection and descriptive statistics was performed with Excel 2010 (Microsoft Inc., Redmond, USA). Data were either presented at the patient level or summarized by means and standard deviations (SD) for the full cohort. AQoL-8D questionnaires were scored with the weighted utility algorithm for SPSS (available at http://www.aqol.com.au/index.php/scoring-algorithms). For the SSQ-12-B questionnaire, scores for ‘speech hearing’, ‘spatial hearing’ and ‘qualities of hearing’ (subscale scores) were calculated as mean value (± SD) over respective items. An overall score was calculated from the subscale scores. APSQ scores for the subsection ‘wearing comfort’, ‘social life’, ‘usability’, and ‘device inconveniences’ were calculated as mean value (± SD) over respective items.

## Results

Patients of all ages (26–85 years) were included in this study (sex: *m* = 7, *w* = 5, *d* = 0). Six patients received a BB implant on the right side, five on the left side. One patient was implanted bilaterally. Ten out of 12 patients had a history of tympanoplasty or stapes surgery. The etiology of hearing loss varied among our patients. Nearly two-thirds of all patients suffered from mixed hearing loss, one patient suffered from an unilateral functional deafness and the rest showed a conductive hearing loss without any inner ear deafness. 7 Patients have had ear surgery due to chronic epi- or mesotympanic otitis before. Recurrent inflammation (recurrent external otitis, recurrent auricular perichondritis) and otosclerosis both were found in two cases. One patient showed an atresia of the external auditory canal.

Bone conduction thresholds of the implantation side and the contralateral ear variied among our cohort. Three patients showed a bone conduction threshold from 10 to 20 dB HL, a threshold from 20 to 30 dB HL was found four times and other four patients had a bone conduction threshold from 30 to 40 dB HL of the implanted ear. Once there was a bone conduction with more than 40 dB HL (patient no. 12, Table 1). The bone conduction threshold of the implanted ear and the contralateral ear deviated in most cases up to 10 dB HL, two patients showed a higher difference of their thresholds (Table1).

### AQoL-8D-questionnaire

The AQoL-8D score for all patients after BB was an overall utility score mean of 0.76 (SD ± 0.17; Min = 0.46; Max = 0.95). The lowest superdimension ‘mental’ utility score in our cohort was 0.2, and the highest was 0.75. The mean of the superdimension ‘mental’ utility scores was calculated at 0.47 (SD ± 0.17).

**Fig. 1:**
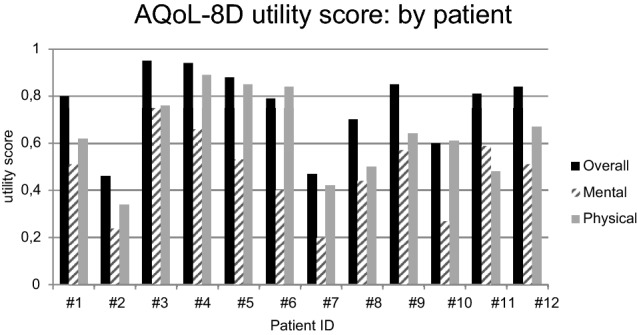
AQoL-8D analysis by patient: Overall utility scores and scores for ‘mental’ and ‘physical’ superdimensions by patient. Overall—overall utility score, Mental—superdimension ‘mental’ utility score, Physical – superdimension ‘physical’ utility score

The mean of the superdimension ‘physical’ utility scores was 0.64 (SD ± 0.18) with a maximum of 0.89. and a minimum of 0.34 (Fig. 1).

**Fig. 2:**
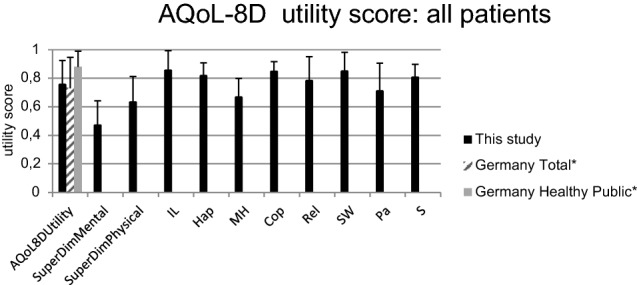
AQoL-8D analysis: Mean values of the cohort in comparison to Germany total and Germany Healthy Public. * Data from “Richardson, J. et al. (2013): Cross-national comparison of twelve quality of life instruments: MIC Paper 7 Germany” [18] IL – dimension ‘independent living’; Hap—dimension ‘happiness’; MH—dimension ‘mental health’; Cop—dimension ‘coping’; Rel—dimension ‘relationships’; SW—dimension ‘self worth’; Pa—dimension ‘pain’; S -dimension ‘senses’, SuperDimMental—superdimension ‘mental’, SuperDimPhysical—superdimension ‘physical’

The dimension ‘senses’ showed a mean of 0.81 (SD ± 0.65). The mean values of all dimension and superdimension utility scores (‘mental’ and ‘physical’) of the AQoL-8D questionnaire are shown in Fig. 2 in relation to data of the German total population and the healthy German population (Germany Healthy Public) [18].

### SSQ-12-B- questionnaire

11 Out of 12 patients reported an improvement in their overall hearing experience (positive overall score). The highest overall score in the evaluation of the hearing situation compared to before BB was + 4.44 and the lowest was – 2.50. Similarly, the subscale scores of ‘speech hearing’, ‘spatial hearing’ and ‘qualities of hearing’ were at least + 1 in 11 of 12 cases.

**Fig. 3:**
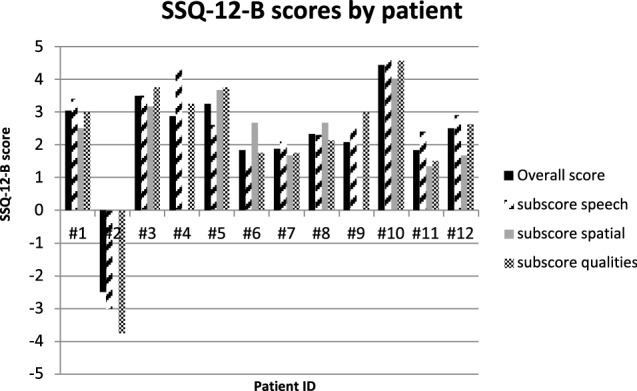
SSQ-12-B analysis by patient: Overall score and subscale scores by patient. subscore speech – subscale score for ‘speech hearing’, subscore spatial – subscale score for ‘speech hearing’, subscore qualities – subscale score for ‘qualities of hearing’

**Fig. 4:**
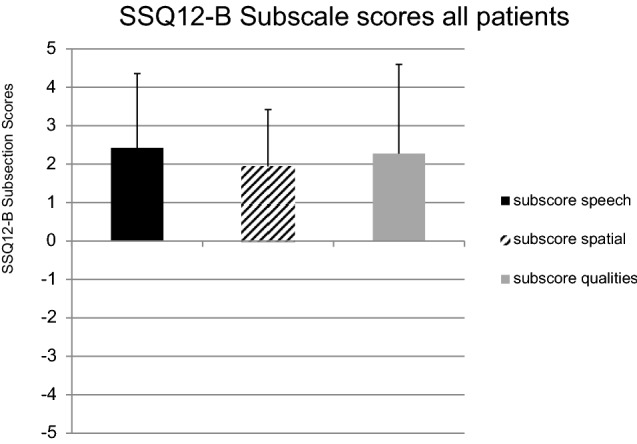
SSQ-12-B subscale analysis: mean of the cohort. subscore speech—subscale score for ‘speech hearing’, subscore spatial—subscale score for ‘speech hearing’, subscore qualities—subscale score for ‘qualities of hearing

A negative overall score (– 2.5) was found once. The subscale ‘speech hearing’ and ‘qualities of hearing’ were rated with – 3 and – 3.75 by patient no. 2. The subscale ‘spatial hearing’ achieved a score of 0 in this case. The results of the SSQ-12-B questionnaire are shown in Fig. 3.The mean subscale score for the item ‘speech hearing’ was + 2.43 (SD ± 1.94), + 1.94 (SD ± 1.48) for ‘spatial hearing’ and + 2.28 (SD ± 2.32) for ‘qualities of hearing’ (Fig. 4).

### APSQ

The mean score of the subsection ‘wearing comfort’ was 3.50 points (SD ± 0.87). The average subsection score for ‘social life’ was 4.17 (SD ± 1.06). The ‘usability’ of the device was rated with a score of 4.23 (SD ± 1.06) and the ‘device inconveniences’ subsection was 4.02 (SD ± 0.71).

**Fig. 5:**
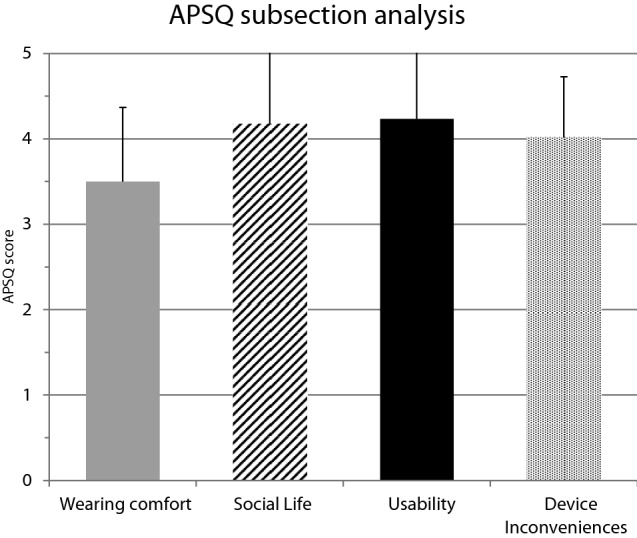
APSQ subsection analysis: mean of the cohort. Wearing comfort – subsection score for ‘wearing comfort’, Social life—subsection score for ‘social life’, Usability—subsection score for ‘usability’, Device inconveniences -subsection score for ‘device inconveniences’

The maximum for ‘wearing comfort’ was 4.33, for ‘social life’ 5.00, for ‘usability’ 4.8 and for ‘device inconveniences’ 4.8. The minimum for ‘wearing comfort’, ‘social life’ and ‘usability’ was 1.0; the minimum for ‘device inconveniences’ was 2.40. Besides one patient, 11 of 12 subsection scores were calculated above a score of 3. The results of the APSQ are shown in Fig. 5 as mean values of the total cohort. 8 out of 12 patients reported an audio processor wearing time of 12 h per day. This value also corresponded to the maximum wearing time of the cohort. The minimum was 0 h per day, while the mean was 10.9 h (SD ± 2.0). None of the patients reported wearing the audio processor at night.

## Discussion

One aim of this study was to determine the quality of life after BB implantation. Mulrow et al. were able to demonstrate a restriction of the quality of life in a study of 472 patients who were affected by hearing loss [1]. The results of our study show that the assessed quality of life of the cohort significantly increased after BB implantation (Fig. 1). According to our data, the BB offers patients suffering from conductive hearing loss, mixed hearing loss or SSD the possibility to increase their perceived quality of life above the average of the overall German population (overall utility score 0.76 vs. 0.73, Fig. 2), maybe this is since hearing rehabilitation is perceived as a particularly strong improvement in quality of life. In comparison, the healthy German population only reported a slightly higher average quality of life (overall utility score 0.88 vs. 0.76, Fig. 2) [18].

Moreover, the results of the AQoL-8D reveal that our patients rated the superdimension ‘mental’ worse than the superdimension ‘physical’ on the overall average (Fig. 2). According to these ratings, the hearing rehabilitation thus seems to have an impact primarily on the physical aspects of our patients’ lives. An interesting fact is that the superdimension dealing with the physical quality of life (superdimension ‘physical’) is composed of the dimensions ‘independent living’, ‘senses’ and ‘pain’. A dimension score ‘senses’ of 0.81 implies a subjective improvement of sensory perception. Thus, a successful hearing rehabilitation can be assumed in our cohort with BB. Skarżyński et al. already propagated that hearing rehabilitation with the BB in 21 patients did not only lead to an audiological improvement, but also to an increase in the subjectively assessed quality of life. In contrast to the questionnaire employed by our group, they used the APHAB questionnaire [19]. Since both instruments led to similar results, our results can be confirmed as valid. In a current study by Garcier et al. (2021) comprising 24 patients the APHAB questionnaire was also used to ensure reproduceable results of an improvement in hearing performance and quality of life after implantation of the BB [20].

The analysis of the SSQ-12-B emphasized a subjective hearing improvement in our BB patients. Our cohort reported an improved hearing experience on average subscale scores (‘speech hearing’ = 2.43, ‘spatial hearing’ = 1.94, ‘qualities of hearing’ = 2.28) after implantation of the BB. 11 out of 12 patients reported positive values for each SSQ-12-B score (Fig. 3). The greatest satisfaction was achieved in ‘speech hearing’ (mean subscale score = 2.43) and ‘qualities of hearing’ (mean subscale score = 2.28), whereas ‘spatial hearing’ showed the least improvement (Fig. 4). In a study by Laske et al. from 2015, the subjective hearing benefit after implantation of a BCI was also determined using the SSQ-B questionnaire. Their positive results in patients’ satisfaction with the implant are in line with our results, whereby ‘spatial hearing’ also achieved the lowest results [21].

Weiss et al. (2016) confirmed this observation concerning the ‘spatial hearing’ using audiometric testing. Patients with SSD were excluded from this measurement. A quantifiable improvement in sound localization after BB implantation could not be determined (no significant change in the mean localization error) [22]. Incongruently, Rahne and colleagues showed an audiological improvement in sound localization (significant reduction of the angle detection error) in a cohort of 11 patients (including 1 patient with SSD) in their 2015 study [23]. The meta-analysis by McLeod et al. may help to clarify the upcoming questions to those controversial results. After a literature review, they concluded that a binaural fitting with a BCI is necessary to achieve an improvement in sound localization [24]. This can also be assumed reviewing our data. In one case of binaural fitting, we found the greatest subjective improvement in ‘spatial hearing’, which is discussed as a difficult and controversial aspect of BCI treatments in the literature [22]. Patient 6, who received a bilateral BB implantation, showed a higher ‘spatial hearing’ subscale score in comparison to the other subscales (Fig. 3). The rating evaluated by patient 6 (‘spatial hearing’ subscale score = 2.67, Fig. 3) was even higher than the average score of the full cohort (mean subscale score = 1.94, Fig. 4). This fact could support the interpretation, that the spatial hearing is best served with a binaural BB. A similar bone conduction threshold of both ears can be seen in patient 6 (Table 1).

Moreover the highest SSQ-12-B-overall-scores by patient can be found at lower bone conduction thresholds of the implanted side and with little difference between the thresholds of both ears (Fig. 3, patient no. 1, 3, 5 and 10).

Regarding to our results of the SSQ-12-B and the AQoL-8D patients with higher scores in the AQoL-8D seem to show also higher scores in the SSQ-12-B (Figs. 1 and 3). This shows that hearing rehabilitation directly influences perceived overall quality of life. Hearing loss does not only have a somatic effect, but also influences other areas of life, such as social life and mental quality of life. Several studies in the past have shown that people with hypoacusis have a higher prevalence of associated comorbidities than people with normal hearing [25–27].

The average ratings of the audio processor in our study (Fig. 5) were consistently high with an APSQ score of at least 3.5 out of 5 (mean subsection score ‘wearing comfort’), especially the ‘usability’ and the ‘social life’ subsection of the audio processor were rated particularly high. Despite the evaluation of the three subsections mentioned above, there was a high level of ‘device inconvenience’ in our cohort (mean = 4.02). In conclusion, despite the general user satisfaction, our patients perceive discomfort with the audio processor. ‘Wearing comfort’ reached a mean of 3.50 (SD ± 0.87), which is low in comparison to the average subsection score for ‘social life’ (mean = 4.17; SD ± 1.06). Still the ‘usability’ of the device was rated with a score of 4.23 (SD ± 1.06). In average, the ‘device inconveniences’ subsection (4.02; SD ± 0.71 outweighed the comfort experienced with the audio processor.

Nevertheless, 8 out of 12 patients reported a wearing time of 12 h per day. The willingness of our cohort to wear the audio processor for many hours a day, despite any device inconvenience, reinforces the conclusion that the gains in quality of life and hearing experience outweighs any device disadvantages. High scores for ‘social life’ and ‘usability’ confirm that assumption. A possible explanation for device inconvenience relates to the practicability of the audio processor in everyday life. Garcier et al. stated that even if the handling and aesthetics of the device was rated positively, the average results decreased especially in wearing the audio processor during sports or at work [20]. The lowest average score results of our study in the subsection ‘wearing comfort’ supports those findings.

Patient no. 2 rated all questionnaires with the lowest scores and reports a deterioration in his subjective hearing experience after BB implantation. In this case about 4 months after the BB implantation (in 2013), a revision surgery (repeated additional skin thinning) was performed. The magnetic fixation of the audio processor had not been sufficient in the obese patient. Furthermore, the patient complained of recurrent pain around the implant. The BB was removed in 2020 at the patient’s request.

Regarding to the bone conduction threshold of patient no. 2 there is an individual inner ear hearing loss of 30 dB HL on the implanted ear. Questioning whether a severe inner ear deafness could have harmed the patient reported outcome, it becomes clear that other patients with similar bone conduction thresholds have rated the questionnaires with better results. In summary there cannot be found a significant pattern between certain bone conduction thresholds of our patients and their ratings in the PROM.

Retrospectively, patient no. 2 can be classified as a non-user (wearing time of 0 h a day). Apart from the fact that generally up to 20% of all patients with hearing aids are non-users [28], special attention should be paid to the regular adjustment of the hearing system. Uncomfortability in the sense of pain occurs in up to 15% of all patients with a hearing aid (of any kind) [29]. That underlines the importance of a strict follow-up. Standardization in the form of a guideline, has been confirmed by Oh et al. among others. According to their study the acceptance of hearing aids was increased and non-user rate was reduced due to the standardization of aftercare [30].

Comparing our results to the recent literature one limitation is the variable time (8 to 48 months) between BB implantation and the survey. However, Table 1 shows that in 10 out of 12 cases this period exceeds at least 1 year. Familiarization and adjustment should be reached at that point.

Another limitation of our study could be seen in the missing correlation of audiological testing, which has been excluded in the sense of PRO [12]. However, PRO is an accepted method to test the outcome regarding to the quality of life with a certain therapy [31–34]. In the case of the bone-anchored hearing aid (BAHA) a number of other investigations proved an enhancement of QoL [35–37]. Badran et al. showed in their BAHA-study already in 2006 the importance of patient’s self-rated QoL to justify a procedure and to reach a better predictive value at the time of preoperative counseling [38]. Han et al. showed 2020 a higher device usage of BB in comparism to BAHA in patients with mixed hearing loss or SSD [39]. As already discussed in this work, the success of hearing aids depends on the patient’s compliance [9, 10]. The high device usage in the investigation of 2020 can be found in our work and therefore, proves the self-reported benefits of BB implantation and a positive PRO. Other recent studies external to Oto-Rhino-Laryngology showed an impact to monitore therapy effects using patient related measurements (PROM) [12, 40]. Monitoring the efficacy of a therapy by PROM should be considered ever then since the optimized use of medical resources in nowadays society gets more and more important [41]. Moreover, improvements of health care and better decision making for future patients and clinicians can be supported by QoL data after a therapy or procedure [42]. We used three different questionnaires, which has so far, to our knowledge, not been utilized before to assess perceived hearing rehabilitation.

Since the impact of the BB in audiometric testing and the effectiveness has already been proven in previous trials [43–46], we focused on comparing the different questionnaires among each other instead of analyzing the questionnaires to audiometric testing results. As Snik et al. reported that audiometric testing can vary within one examination of a patient [47], we also saw a great variance in our patients’ audiometric test evaluations. This fact even supports our decision to omit audiometric testing in our study. Moreover, due to the inhomogeneity of diseases of our cohort, the respective patient history the comparison of our patients’ audiometries seemed not be feasible in a meaningful way. Different investigators during audiometric testing can be seen as another bias.

## Conclusion

In summary, our results show an appropriate perceived hearing rehabilitation with the BB. The evaluation of quality of life, the decrease of the self-reported auditory disability and patients’ satisfaction can confirm the BB as an adequate therapy for auditory rehabilitation. Especially the improved quality of life and the outstanding results in speech hearing of the cohort after BB implantation can lead to an increase of social life satisfaction. Spatial hearing showed the least improvement with BB, which is also discussed in recent studies and seems to be served best in the case of a binaural BB implantation.
